# The Gender Lens: Development of a learning aid to introduce gender medicine 

**DOI:** 10.3205/zma001094

**Published:** 2017-05-15

**Authors:** Simone Weyers, Anja Vervoorts, Nico Dragano, Miriam Engels

**Affiliations:** 1 Heinrich-Heine-Universität Düsseldorf, Medizinische Fakultät, Centre for Health and Society, Institut für Medizinische Soziologie, Düsseldorf, Deutschland; 2 Heinrich-Heine-Universität Düsseldorf, Medizinische Fakultät, Dekanat, Düsseldorf, Deutschland; 3 Heinrich-Heine-Universität Düsseldorf, Medizinische Fakultät, Institut für Medizinische Soziologie, Düsseldorf, Deutschland

**Keywords:** sex- and gender-sensitive medicine, gender medicine, teaching material

## Abstract

**Background and aim:** Gender medicine takes into account biological and social differences between men and women in terms of prevalence and course of disease, diagnosis and therapy. Medical students should be made aware of this in the early stages of medical education. However, there is hardly any teaching material currently available. This article presents the adaption and first use of the German “Gender Lens,” a tool to introduce gender medicine to medical students.

**Method: **The original Canadian ”Gender Lens Tool” was translated into German, tested by (n=5) teachers and adapted based on current scientific concepts. The instrument was applied and evaluated using qualitative methods in a student focus group (n=4). It was then piloted in a cohort of fourth-semester students (n=247) in a seminar addressing gender medicine. These experiences were evaluated using quantitative methods.

**Results: **The German translation of the Gender Lens offers students a framework with which to analyze sex and gender differences in terms of the “prevalence, diagnosis, course, therapy and prevention” of a specific disease. Furthermore, it enables a refined search for causes such as “biological disposition, attitudes and behaviors, family and social networks, occupational and material circumstances and experiences with the health care system.” Recommendations were received from the student groups regarding teaching methods. Male and female fourth-semester students agreed that the Gender Lens is useful as an introduction to gender medicine.

**Discussion: **Initial experiences with the Gender Lens adapted for the German curriculum suggest that such a learning aid can contribute to raising awareness of gender medicine in medical students.

## Zusammenfassung

**Hintergrund und Ziel: **Gendermedizin berücksichtigt biologische und soziale Unterschiede zwischen Männern und Frauen bei der Häufigkeit und dem Verlauf von Erkrankungen, ihrer Diagnostik und Therapie. Studierende der Medizin sollten in der Lehre frühzeitig mit solchen Unterschieden vertraut gemacht werden. Hierzu gibt es bisher jedoch kaum Unterrichtsmaterial. Im Beitrag wird die Adaption und erste Erprobung der „Geschlechterbrille“ vorgestellt, einem deutschsprachigen Instrument, um Medizinstudierende mit der Gendermedizin vertraut zu machen.

**Methode: **Das kanadische Original „Gender Lens Tool“ wurde zunächst wortgetreu übersetzt, dann von (n=5) Dozent/innen exemplarisch durchlaufen und im Hinblick auf die wissenschaftliche Terminologie adaptiert. In einer studentischen Fokusgruppe (n=4) wurde die Geschlechterbrille im Rahmen eines Gruppeninterviews mit Hilfe qualitativer Methoden erprobt und bewertet. In einer Gesamtkohorte von (n=247) Studierenden des vierten Semesters wurde sie dann zur Pilotierung im Seminar zur Gendermedizin eingesetzt und mit Hilfe quantitativer Methoden beurteilt.

**Ergebnisse: **Die aus dem Prozess resultierende Geschlechterbrille bietet eine Struktur, mit der Studierende in Bezug auf eine spezifische Erkrankung Geschlechterunterschiede in „Häufigkeit, Diagnose, Verlauf, Therapie und Prävention“ analysieren können. Sie ermöglicht weiterhin eine strukturierte Recherche nach den Hintergründen von Geschlechtsunterschieden entlang der Dimensionen ‚biologische Unterschiede, Einstellungen und Verhaltensweisen, Familie und soziales Netzwerk, Arbeitsbedingungen, materielle Bedingungen und Erfahrungen mit dem Gesundheitssystem‘. In den studentischen Fokusgruppen ergaben sich Hinweise zum didaktischen Vorgehen. Die männlichen und weiblichen Studierenden des vierten Semesters stimmten zu, dass die Geschlechterbrille zur Einführung in die Gendermedizin sinnvoll ist.

**Diskussion:** Erste Erfahrungen mit der für den deutschsprachigen Unterricht adaptierten Geschlechterbrille legen nahe, dass sie die Studierenden der Humanmedizin bei der Sensibilisierung für das Thema unterstützten kann.

## 1. Introduction

Clear differences exist between the genders in regard to life expectancy and disease. On average, women live approximately five years longer than men (82.7 vs. 77.7 years) [1]. Further reflecting this disparity, premature mortality in men aged 30 to 64 is twice as high as it is in women. This excess mortality can be explained predominantly by accidents, suicide, and diseases such as heart attack, lung cancer and cirrhosis of the liver [2]. When it comes to pathological processes, some differences are more disadvantageous for men, others more for women. For instance, coronary heart disease develops earlier in men than in women. In contrast, women are more likely than men to be treated for a mental disorder or to go into early retirement as a result of one [3].

Disparities in life expectancy, prevalence and severity of disease have various causes. First, the biological differences between men and women must be considered since these involve bodily functions and etiopathogenic processes [4]. However, biological factors alone do not account for gender differences in health and life expectancies. In addition to biological determinants, social determinants also play a role in supporting health or causing disease in men and women. For example, men and women differ in their health-relevant lifestyles, their living and working conditions, and how they utilize health care [5]. One study of more than 11,000 Catholic monks and nuns was able to show that the life expectancies for men and women is very nearly even when they experienced similar living and working conditions [6].

Accordingly, medicine should take the biological and social differences between men and women and their significance concerning disease, prevention, diagnostics and therapy into account to offer targeted medical care. Medical education can serve a critical role in that it teaches students to recognize biological and socially constructed gender differences and to apply this knowledge to their practice of medicine [7]. Medical students, however, are often unaware of the significance of gender to pathogenesis and medical treatment [8]. A survey of medical students regarding the importance of gender-sensitive medicine demonstrated that this was frequently mistaken for gender equality and employment issues such as equal pay or career opportunities [9].

Patient-centered medical science is anchored in many medical curricula. However, there are hardly any curricular concepts or teaching materials incorporating gender medicine into medical education [10]. In response to this need, the project “Männer, Frauen und Medizin” (Men, Women and Medicine) took place in 2014. Its goal was to develop a strategy for introducing gender-sensitive medicine into the Düsseldorf medical curriculum.

First, an analysis of the current situation was undertaken focusing on the topics in the international competency catalogue on gender differences in health care (APGO) [11]. This revealed that practically all competencies are taught by at least one department of the medical school. When questioning the students, however, a discrepancy emerged between what is taught and what is learned. Primarily in the preclinical study phase students are hardly concerned about gender-sensitive medicine. An earlier study describing the study program at the same university in question here showed that most medical students had little contact with the topic of gender-sensitive medicine [12].

It is evident that, above all, there is a need to raise awareness among medical students so that they are better able to discern the existing curricular content on biological and social gender differences. Consequently, the curricular development focused on introducing a tool to sensitize students during the early study phase. Literature research on relevant learning aids yielded two possibilities: a medical sociology exercise on gender habitus [13] and the “Gender Lens Tool” from the Canadian Gender and Health Collaborative Curriculum Project. The latter serves to encourage detailed consideration of the gender differences concerning specific clinical pictures and their causes [14]. Since the Gender Lens Tool appeared to be a promising strategy for encountering gender medicine for the first time, it was selected for refinement.

The aim of this paper is to describe the development of the Gender Lens for the German-speaking countries and to present the results of its first use in the context of teaching medical sociology.

## 2. Project description and results

### 2.1. Translation and adapted version

**Figure 1:**
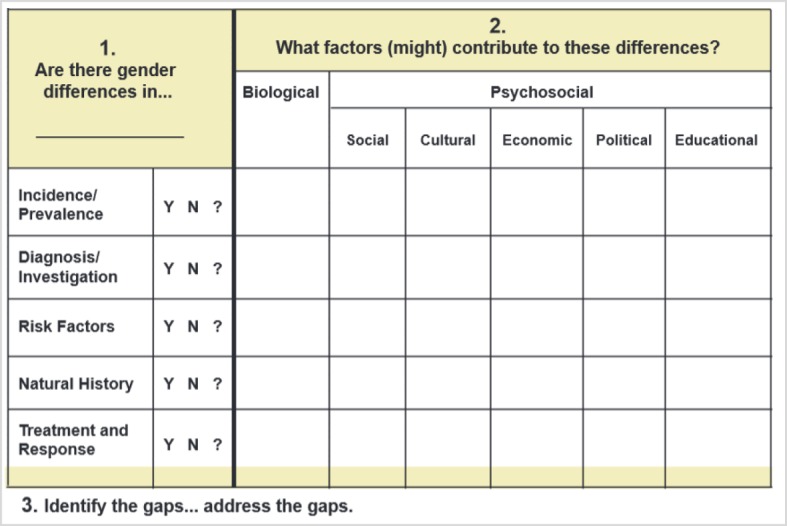
Original Gender Lens Tool [14]

The Canadian Gender Lens Tool starts with a vertical axis asking if there are differences in the occurrence of a specific disease (incidence/prevalence, diagnosis/investigation, risk factors, natural history, treatment/response). The second axis searches for possible factors or causes for these differences (biological vs. psychosocial: social, cultural, economic, political, educational) (see figure 1 (Fig. 1)).

This tool was initially translated verbatim and presented to a group of university lecturers (n=5; 2 male/3 female). As an experiment, “depression” was taken as an example and worked through, then discussed and adapted based on German medical terminology and the prevailing German discourse.

In the course of this, “risk factors” was deleted from the vertical axis since this aspect is better situated on the axis dealing with causes. The aspect of “prevention” was inserted instead since there are distinct differences between men and women in the utilization of prevention [5], [15], and prevention should be placed on the same level as therapy. This resulted in a vertical axis listing differences in the following areas: incidence/prevalence, diagnosis, course of disease, therapy and prevention.

On the axis listing the causes no distinction was made between biological and psychosocial since this dichotomization is not an exhaustive one in our opinion. Instead, drawing on the model of social determinants [16], a distinction was made between the following causes: biological causes, attitudes and behaviors, family and social network, work conditions, material conditions and experience with the health care system.

**Figure 2:**
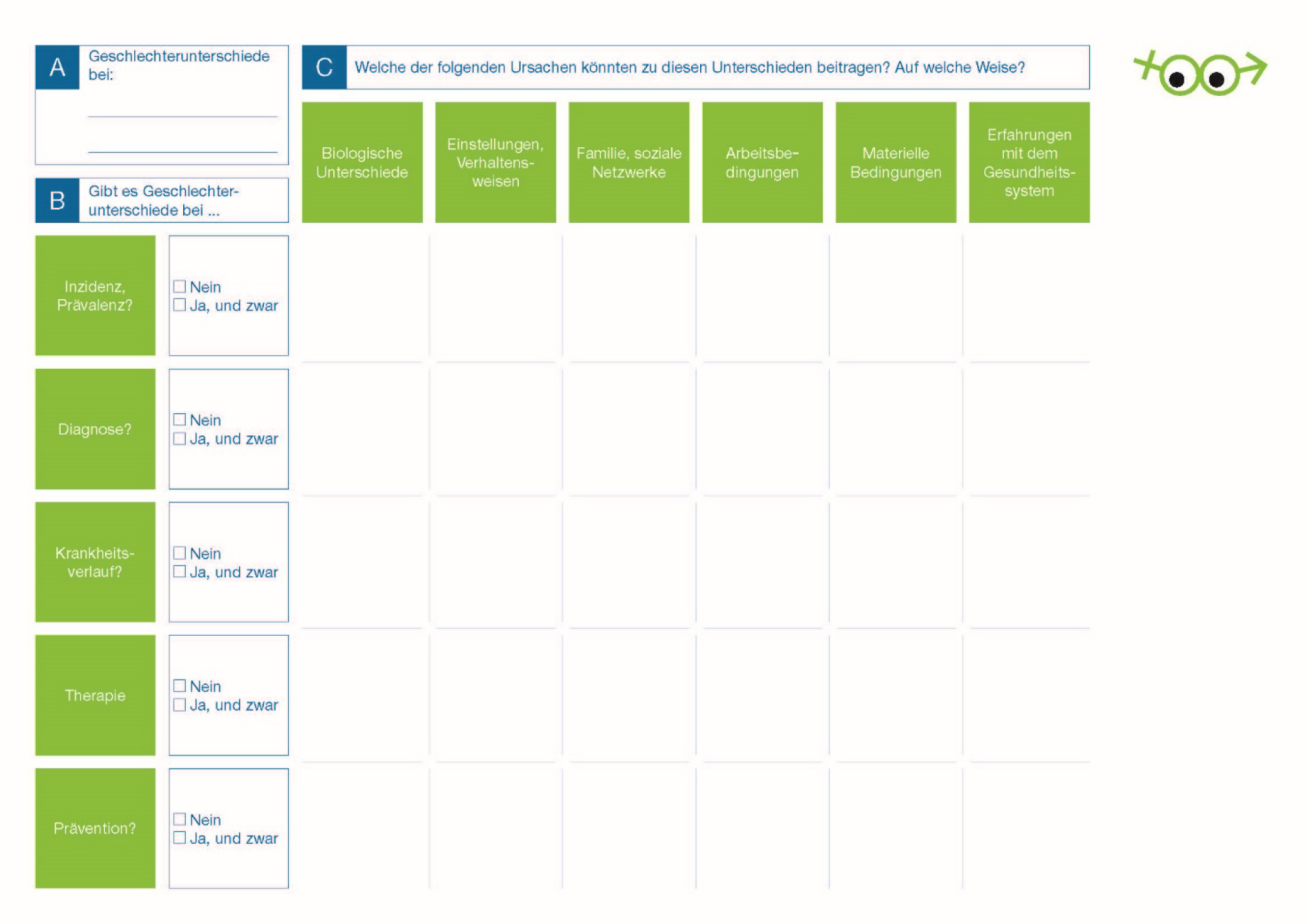
Modified Gender Lens

The result is a tool that can be applied in three steps (see figure 2 (Fig. 2)). First, a disease is selected (cell A). Then it is ascertained whether the disease shows gender differences in terms of incidence/prevalence, diagnosis, course of disease, therapy and prevention (column B). Sample questions relating to a specific disease include:

- Are men more often affected by a disease than women, or vice versa?
- Do the disease symptoms vary between men and women, and how does this affect diagnosis?
- Are men affected differently by pathogens than women?
- Do men show a different adherence to therapy than women?
- Do women participate more often in screening programs than men?

In a third step, hypotheses can be posed about which causes could contribute to the gender differences (cell C). Example questions regarding a specific disease include:

- Does the female menstrual cycle affect the efficacy of medications?
- Do boys and girls differ in their attitudes toward disease prevention?
- Do men and women experience differing degrees of support from family members when coping with disease?
- Do the working conditions of men promote the development of disease to a greater extent than the working conditions of women?
- Do men and women differ in regard to economic resources for managing disease?
- Do men report symptoms differently than women?

#### 2.2. Piloting in the student focus group

The tool was first piloted in a group of medical students within the context of a group discussion. All students enrolled in the university’s medical school received an email and were personally invited to participate. Four students were recruited (1 male/3 female). As an incentive to attend, they were each given a gift certificate to buy books.

Following the group discussion on the relevance of gender medicine and the way it is currently taught at the University of Düsseldorf, the students were introduced to the Gender Lens. The assignment was to apply the Gender Lens to “depression” and then critically evaluate the tool’s value and practicability. The discussion was recorded and transcribed verbatim. The data were analyzed by two impartial people using inductive content analysis [17]. Categories were created from the text material using an open coding procedure, and the statements were classified and abstracted. The following points became visible:

##### Positive criticism

**Personal initiative**: Students reported that the tool animated them to pursue targeted research and enter discussions and fostered independent study of the topic.

**Learning process: **Students prefer the seminar as the learning setting in which to explore aspects of gender medicine. Initially, courses in the clinical subjects were cited as suitable settings for using the Gender Lens; however, later students were of the opinion that a separate course unit during the early semesters would be more meaningful. Using the tool, students could practice viewing selected clinical pictures through the Gender Lens and would then be open to aspects of gender during the later clinical phase of study.

**Content: **Students found it positive that the Gender Lens helps to (re-)illuminate a medical topic from the perspective of gender medicine. By doing this, new aspects regarding the differences between men and women become clear, for instance, in the areas of prevention and therapy. As a result, the tool helps to create an informed awareness for future interactions with male and female patients.

##### Suggestions for improvement

**Assignment: **Students indicated that the task they were asked to perform was not communicated clearly. This referred to the sequence of the steps to be carried out. Also, the question was raised about sources of information, meaning should students cite their own ideas or provide available scientific evidence regarding the different mortality rates and causes of morbidity.

**Insufficient space: **Although the tool’s table did provide a framework for classifying the differences and causes regarding morbidity, the students found themselves pressed for space to write down information. The students desired more room to list differences and formulate key phrases related to the causes.

**Figure 3:**
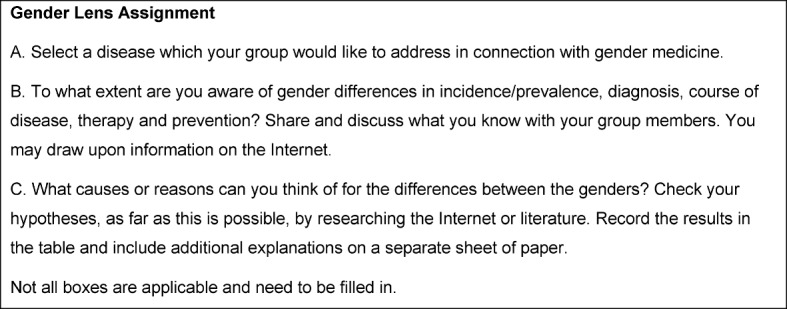
Assignment using the Gender Lens

In response to student suggestions for improvement, detailed instructions for the assignment were formulated (see figure 3 (Fig. 3)), the steps of the assignment were designated with A, B, and C, and an additional section was included for listing and describing morbidity differences. Additional keywords regarding causes could be listed on a separate sheet of paper. The grid, however, was retained since we are of the opinion that it illustrates the many facets of gender medicine and sharpens the critical eye.

#### 2.3. Piloting in the medical sociology core curriculum

After its initial use in a small-group setting, the Gender Lens was used for the entire cohort of fourth-semester medical students in the context of a 90-minute seminar addressing the topic of men, women and medicine. The learning objectives for this session aimed at enabling students to describe and analyze gender differences as they relate to pathological processes and to explain the basic principles of gender medicine. To meet these objectives, the lecturer first outlined and explained the differences in premature mortality and the main causes of death as well as the differences in epidemiologically relevant diseases. Based on the instructions in box 1, students then worked through the Gender Lens using the example of coronary heart disease (CHD) (see figure 3 (Fig. 3)). Students were given a paper printout. Given the limited time, students focused their attention on question C to hypothesize about gender differences in the incidence and prevalence of CHD. In the final part of the session, the lecturer presented evidence on gender differences in the groups of causes which were then juxtaposed with the hypotheses students had posited directly before. At the close of the seminar, the students evaluated the Gender Lens using a standardized anonymous questionnaire. Using a six-point Likert scale, they indicated the extent to which they agreed or disagreed that the Gender Lens is a valuable and meaningful introduction to gender medicine, is clearly organized and structured, easy to use and encourages reflection.

In the analysis the mean and standard deviation were calculated for each item. The analyses were carried out for the entire sample of 247 medical students and again separately for men (78) and women (130; 39 missing information on sex). Any differences between the evaluations of men and women were identified using the t-test for independent samples. The results are presented in table 1 (Tab. 1).

With values between 2.14 and 2.32, students in the overall sample agreed, on average, with the statements above regarding the Gender Lens. In terms of the conventional academic grading scale this rating corresponds to a score of “good.” The analysis revealed no differences in the way men and women evaluated the tool.

## 3. Discussion

### 3.1. Summary

The learning aid described here is based on the Gender Lens Tool from the Canadian Gender and Health Collaborative Curriculum Project. It was translated into German as described above, piloted and adapted, and evaluated by two groups of students.

The results give the initial impression that offering such a reflective unit during the preclinical study phase can be a valuable strategy for introducing gender medicine into the medical curriculum. Medical sociology can serve as the setting for such a course unit since the pertinent content is part of its curriculum, and a required course will reach all students in a cohort.

#### 3.2. Limitations

Initial experiences in the core curriculum do show that careful attention must be paid so that no stereotypes are reinforced or perpetuated with the exercise. Assumptions that are made ad hoc in the first steps of the assignment can reveal themselves to be false. To support and encourage this process, evidence-based material and sources need to be compiled in a follow-up step.

The perspectives of teaching staff should be included in the further refinement of this tool, in particular medical lecturers. Including teachers enables verification of the tool’s relevance as an introduction to gender medicine and its alignment with the curricular content of the clinical study phase.

#### 3.3. Outlook

The National Competency-based Catalogue of Learning Objectives for Undergraduate Medical Education (NKLM) states that students should be able to assess the health of male and female patient groups and to identify and give sufficient consideration to gender-based concerns when communicating with patients or providing therapy and prevention [www.nklm.de]. The Gender Lens can be used to accomplish this in the German-speaking countries. In the early study phase, medical students can be introduced to gender medicine using an example ailment that allows them to explore its implications for the two genders. Students will then in the later study phase be open to the different clinical aspects of prevention, diagnosis and therapy regarding men and women. The tool itself can be used in a variety of ways, in single sessions or over a sequence of course sessions. The tool is meant to aid students in better identifying and absorbing existing course content on biological and sociological gender differences. This is an important step in the education of future physicians who, even after completing their medical studies, are able to internalize a gender-sensitive perspective in their practice of clinical medicine.

## Acknowledgements

We wish to thank the sponsors and coordinators of the KomDiM program which served as the basis for the curricular project on men, women and medicine; Dr. Anna Day of the Canadian Gender and Health Collaborative Curriculum Project for allowing us to use Gender Lens Tool; the medical students for the dedication and commitment they exhibited in the evaluation; Benjamin Brinkmann for the visual layout of the German Gender Lens.

## Funding

Funding was received from the Federal Ministry for Education and Research (ID no. 01PL11083C).

## Informed consent

The process for gathering informed consent from the participants was approved by the Ethics Commission of the University of Düsseldorf Medical School (Study number 5012).

## Competing interests

The authors declare that they have no competing interests.

## Figures and Tables

**Table 1:**
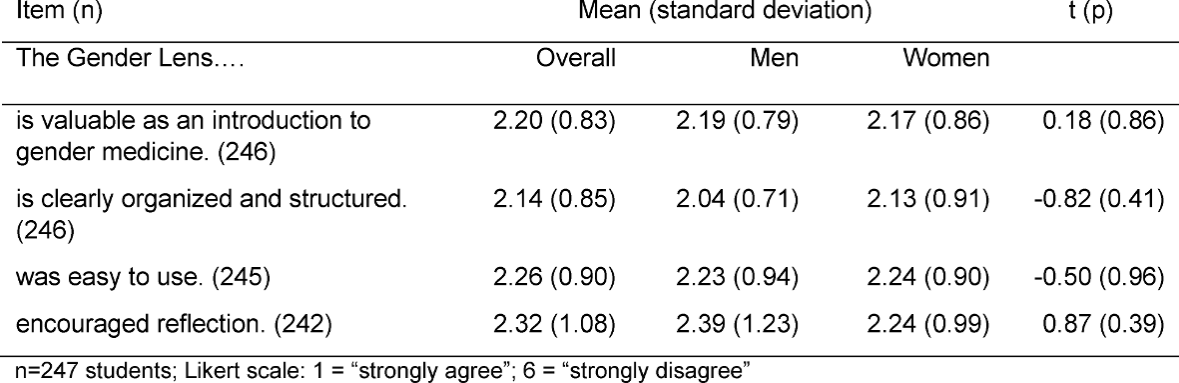
Evaluation of the Gender Lens
